# Is American Bioethics Lost in the Woods?

**DOI:** 10.1371/journal.pmed.0020121

**Published:** 2005-04-26

**Authors:** Michael Cook

**Affiliations:** **1**BioEdgeAustralasian Bioethics InformationMelbourne, VictoriaAustralia

The debate between a libertarian bioethicist and a communitarian bioethicist [1] illustrates why American bioethics is becoming increasingly marginalised and irrelevant to the democratic society that it intends to serve.

Both participants in the debate, Arthur Caplan and Carl Elliott, explicitly reject the notion of “human nature” as a foundation for bioethics. But without human nature, on what grounds can advances in biomedical knowledge be called good or bad, right or wrong, or even harmful or beneficial? Clearly Caplan and Elliott have to accept something as a touchstone of their bioethical discourse, or it will lapse into windy incoherence. Although they approach it from different angles, this benchmark is informed consent, with Elliott placing the stress on “informed” and Caplan on “consent”.

As a result, their lively disagreement over enhancement technology is just verbal sparring and not a battle of ideas. Caplan believes that the consumer-patient is sufficiently mature to weigh up the dangers; Elliott is more sceptical. Neither appears to think that it makes any sense to argue that technology should be suited to human nature. This belief seems to be widespread in the bioethics community. Ruth Macklin, a bioethicist at Albert Einstein College of Medicine, argued recently, for instance, that “human dignity” is an empty and meaningless concept [2].

However, academic discourse has failed to dislodge from the heads of the hoi polloi the conviction that the starting point of ethics is not consent but happiness. The man in the street, the ultimate consumer of bioethics, still believes in human nature. The notion that human dignity is meaningless would be regarded by nearly all Americans as not merely absurd but reprehensible.

What I find odd in the writings of many bioethicists is that they skirt around the question that the average person wants to ask: will this enhancement make me happy in a deeply satisfying and fulfilling way? He or she is much less interested in whether all the boxes on the informed consent form have been ticked properly.

Consequently, as the Caplan–Elliott bunfight demonstrates, bioethicists are now reduced to arguing that human enhancement is good if people want it—even if they want it mainly because powerful commercial interests have persuaded them to, even if it is weird and kinky, even if it won't make them happy. Elliott's fascinating book *Better than Well* [3] is evidence that exercising a right to enhancement still leaves many lives hollow and unhappy. Sooner or later people will ask why they hadn't been warned, and a lot of bioethicists will be looking for jobs.
